# Erosion reduces soil microbial diversity, network complexity and multifunctionality

**DOI:** 10.1038/s41396-021-00913-1

**Published:** 2021-03-12

**Authors:** Liping Qiu, Qian Zhang, Hansong Zhu, Peter B. Reich, Samiran Banerjee, Marcel G. A. van der Heijden, Michael J. Sadowsky, Satoshi Ishii, Xiaoxu Jia, Mingan Shao, Baoyuan Liu, Huan Jiao, Haiqiang Li, Xiaorong Wei

**Affiliations:** 1 State Key Laboratory of Soil Erosion and Dryland Farming on the Loess Plateau, Northwest A&F University, Yangling, Shaanxi China; 2 CAS Center for Excellence in Quaternary Science and Global Change, Xi’an, Shaanxi China; 3 College of Natural Resources and Environment, Northwest A&F University, Yangling, Shaanxi China; 4 BioTechnology Institute, University of Minnesota, St. Paul, MN USA; 5 College of the Environment and Ecology, Xiamen University, Xiamen, Fujian, China; 6 Department of Forest Resources, University of Minnesota, St. Paul, MN USA; 7 Hawkesbury Institute for the Environment, Western Sydney University, Penrith South DC, NSW Australia; 8 Department of Microbiological Sciences, North Dakota State University, Fargo, ND USA; 9 Agroscope, Department of Agroecology & Environment, Zürich, Switzerland; 10 Department of Plant and Microbial Biology, University of Zürich, Zürich, Switzerland; 11 Department of Soil, Water, and Climate, University of Minnesota, St. Paul, MN USA; 12 Key Laboratory of Ecosystem Network Observation and Modeling, Institute of Geographic Sciences and Natural Resources Research, Chinese Academy of Sciences, Beijing, China; 13 University of Chinese Academy of Sciences, Beijing, China

**Keywords:** Soil microbiology, Microbial ecology

## Abstract

While soil erosion drives land degradation, the impact of erosion on soil microbial communities and multiple soil functions remains unclear. This hinders our ability to assess the true impact of erosion on soil ecosystem services and our ability to restore eroded environments. Here we examined the effect of erosion on microbial communities at two sites with contrasting soil texture and climates. Eroded plots had lower microbial network complexity, fewer microbial taxa, and fewer associations among microbial taxa, relative to non-eroded plots. Soil erosion also shifted microbial community composition, with decreased relative abundances of dominant phyla such as Proteobacteria, Bacteroidetes, and Gemmatimonadetes. In contrast, erosion led to an increase in the relative abundances of some bacterial families involved in N cycling, such as Acetobacteraceae and Beijerinckiaceae. Changes in microbiota characteristics were strongly related with erosion-induced changes in soil multifunctionality. Together, these results demonstrate that soil erosion has a significant negative impact on soil microbial diversity and functionality.

## Introduction

Soil erosion has been identified as one of the greatest challenges for soil health and sustainable development [1–4]. Soil erosion has been accelerated by intensive human activities and extreme climate events [3–5]. Erosion has impacted ~84% of world land surfaces and has led to the degradation of >33% of Earth’s soils [5]. It has been predicted that by 2050 degradation will increase up to 90% [6], severely threatening human welfare [4]. Erosion results in the loss of fertile topsoil and nutrients, which support microbial habitats and soil ecosystem services, leading to reduced productivity [1, 7]. Current soil erosion events are projected to result in a 50% loss in crop yields [6]. Moreover, erosion has profound influences on the environment by affecting greenhouse gases and the emission of agricultural pollutants [8–13]. Given its impact on world-wide sustainable development, the United Nations initiated the Stop Soil Erosion, Save Our Future campaign as the topic of the 2019 World Soil Day [6].

Multiple ecological functions occur simultaneously rather than individually. Therefore, use of integrative measure of multiple functions (i.e., multifunctionality, including nutrient cycling, decomposition, primary production, climate regulation, etc.) would increase our ability to understand and predict the services that the soils and the ecosystems provide and how such services respond to biodiversity and environmental changes [14–17].

Soil microbiota are fundamental to the functioning of ecosystem and soil ecological processes, regulating energy flow and mass fluxes, and mediating the response of soil ecosystem to anthropogenic disturbances and environmental changes [15, 18–22]. The composition, structure and functionality of soil microbiota are sensitive to changes in soil environments [23–28]. Any changes in soil microbiota would also have important broad environmental impact by influencing greenhouse gas emissions and carbon and nutrient cycling [29, 30]. Given the importance of soil microbial diversity for ecosystem multifunctionality [15, 31–33], they must be considered when examining the mechanisms behind the response of terrestrial ecosystems to erosion.

The soil microbial world is incredibly diverse with tens of thousands of species members in one gram of soil [18, 34], and diversity is often not adequate to understand microbiome functioning. Associations among individual microbes, sometimes examined via co-occurrence networks, and their functional groups can reveal ecological relationships based on resource availability and environmental heterogeneity [35, 36]. Although co-occurrence network analysis may not always indicate true biotic associations [37], it can help understand the complexity of microbiomes and how such complexity might change in response to environmental factors and how microbe-microbe associations might have implications for ecosystem functioning [23, 36, 38]. Network analysis can also reveal why some microbial groups consistently occur together or whether certain microbial taxa are more important for maintaining network structure. The complexity of co-occurrence network can be assessed by relevant network scores such as degree, clustering coefficient and modularity, which indicate the connectivity among operational-taxonomic units (OTUs). A pertinent question is how soil microbiome complexity, as indicated by network connectivity, changes in response to soil erosion. This is particularly important because recent studies have shown that network connectivity can have important implications for microbiome stability and ecosystem multifunctionality [33]. In addition, keystone microbial taxa are highly connected taxonomic groups that individually, or in a guild, exert a significant influence on microbiome structure and functioning irrespective of their abundance across space and time [39]. These taxa may confer greater biotic connectivity to the community and can be indicators of community shifts [40]. Since a loss of keystone taxa can negatively affect network connectivity, identifying such taxa across erosion levels can yield insights into the impact of erosion, with subsequent implications for microbiome functioning and ecosystem multifunctionality.

The composition and functionality of soil microbiota are regulated by soil abiotic characteristics including pH, texture, and nutrient and moisture availabilities [14, 27, 41], which are likely to be sensitive to soil erosion. Erosion has been shown to negatively impact microbial biomass, abundance, and composition by altering natural soil characteristics and removing vegetation protection [42–44]. In turn, all of these changes may alter the turnover and availability of soil nutrients and soil functionality. Despite their obvious importance, however, it is still unclear how soil erosion and soil degradation induced by erosion alters the structure and function as well as the associations among microbes. In order to restore eroded environments, it is essential that we gain a mechanistic understanding of biotic and abiotic changes induced by erosion and related soil degradation [15, 18–22].

Here we evaluated the response of soil microbial community to erosion by examining how the diversity, composition and network complexity of soil microbiomes differ between non-eroded and eroded plots with varying erosion intensities. We also determined whether erosion-induced changes in microbial communities are associated with soil multifunctionality. We hypothesized that erosion would negatively impact soil microbial diversity and network complexity. To test this hypothesis, we established two independent studies in markedly different systems/sites to examine whether responses would be similar in such different contexts. The study was conducted at Nenjiang in Heilongjiang Province and at Fuxian in Shaanxi Province of China (Fig. S1). These two sites are separated by a distance of 2500 km but represent two important soil erosion regions in China, and have contrasting soil texture (clay loam vs. sandy loam), climates (mean annual temperature of 0.4 vs. 9.0 °C) and erosion history (>50 years vs. 27 years) [45–47]. At both sites, we selected plots comprising four erosion intensities (i.e., non-eroded soils, lightly eroded soils, moderately eroded soils, and heavily eroded soils) to compose an erosion gradient. The vegetation was the same across the soil erosion gradient at each site (i.e., agroecosystem, with maize and soybean for >50 years at the Nenjiang site and, bare land without any crop for 27 years at Fuxian).

At each site we measured a range of soil physical, chemical, and microbial properties, soil functional parameters, and generated a soil multifunctionality index. We also quantified the diversity and composition of soil bacteria by using 16 S rRNA gene amplicon sequencing. This date was used to construct co-occurrence networks to explore the associations among microbes and identified microbial keystone taxa. Lastly, we linked these parameters to soil functions to test whether microbial community characteristics were related to soil quality and soil erosion. We demonstrated that soil erosion reduced soil multifunctionality and bacterial diversity as well as network complexity and associations among microbial taxa. Moreover, changes in microbiota characteristics were positively related with erosion-induced changes in soil multifunctionality.

## Materials and methods

### Study sites and soil sampling

To examine erosion-induced changes in soil multifunctionality and microbiological properties, two sites were selected at Fuxian (Shaanxi Province) and at Nenjiang (Heilongjiang Province), China. The Fuxian and Nenjiang sites represent the two important soil erosion regions in China, i.e., the Loess Plateau and Northeast China black soil regions, respectively. The two regions together accounted for about 51% of China’s soil erosion regions. Soil erosion in the Loess Plateau region occurs in steep and short slopes, with slope degrees of >15° and a length ~200 m. Soil erosion in Northeast China region occurs on gentle but long slopes, with slope degrees of 1–5° and a length ~2 km.

The soils at Fuxian are loess soils, which corresponds to Calcic Chernozems in FAO taxonomy. The clay content is 11–21%, and the texture is sandy loam. The Nenjiang site has a black soil, which corresponds to Luvic Phaeozem in FAO taxonomy. The clay content is 25–35%, and the texture is clay loam. The climate at Fuxian is characterized by a warm temperate monsoon climate, with a mean annual temperature (MAT) of ~9.0 °C and the mean annual precipitation (MAP) of 577 mm. The climate at Nenjiang is characterized by a cold and semiarid climate, with a MAT and MAP of 0.4 °C and 500 mm, respectively.

In the harvest season of 2017, soil samples were collected from each site. At Nenjiang, we established our sampling plots in a maize (*Zea mays L*.) field (with a width of 260 m and length of 900 m) that was converted from forests more than 50 years ago for agricultural production. The site was in a small watershed in Nenjiang farm (48°59′-49°03′N, 125°16′-125°21′E). The soils were mechanically tilled to 20–30 cm depth every year. Details of the study slope were previously described Zhang et al. [45] and Li et al. [46]. In early October 2017, we established two sampling transects along the slope to collect soil samples. At Fuxian, we selected two cultivated slopes converted from forest in a small watershed (109°11′E, 36°05′N). The two slopes have a width of 13 m, and length of 79 and 83 m, respectively. These two slopes were established in 1989 to simulate soil erosion in cultivated slopes [47]. The soils in the cultivated slopes were tilled to 20-cm-deep before rain season (May) with tillage method widely used in the Fuxian region. The slopes did not receive any fertilization, nor planted with any crop since their establishment. The weeds in the slopes were removed by hand. In late September 2017, we conducted soil sampling in the two slopes.

The erosion usually occurs in slopes while the intensity varies with positions along slope, usually increasing with the degree of slope and the distance from upper slope. Four positions along each of the two slopes/transects were selected at both the Fuxian and Nenjiang sites to represent various soil erosion intensities, i.e., non-erosion (E0), lightly erosion (EL), moderately erosion (EM), or heavily erosion (EH). The erosion modulus in E0, EL, EM and EH sites were <500, 500–2500, 2500–5000 and 5000–8000 t km^−2^ a^−1^, respectively.

In this study, we assumed that soils in all the sampling plots at each slope were the same before erosion, and that any variations in soil properties and microbiota parameters among erosion treatments could be ascribed to soil erosion. The slopes at Nenjiang were planted with the same crops (maize or soybean over the 50 years) and the slopes at Fuxian did not have any crop, the effects of erosion across the erosion gradient within the same site were not affected by vegetation or land use variation. Moreover, the textural composition (clay, silt and sand) and pH in deep soils (30–50 and 50–70 cm for Nenjiang, 20–40 and 40–60 cm for Fuxian) were similar among the four positions in each slope/transect (Fig. S2). Given that soil properties in deep soils were minimally affected by erosion, these similarities suggest that the soils along the slope/transect were similar before erosion, supporting our assumption.

We established three plots (10 × 10 m for Nenjiang, and 2 × 2 m for Fuxian) in each of different slope positions for soil sampling, resulting in a total of 24 samples per site (e.g., 2 transects/slopes × 4 positions × 3 plots). The sampling size was greater at Nenjiang than Fuxian because the size of the slope was greater at Nenjiang (260 m by 900 m) than Fuxian (13 m by ~80 m). In each sampling plot, three soil samples were collected from the 0–20 cm depth, with a 5.0 cm diameter sterilized soil auger. Samples were combined as a composite sample for each plot and were used to measure soil functional metrics as well as soil microbiota.

### Soil analyses

For this study, 13 soil variables were measured, of which 12 were used to assess the overall functioning of the soil (expressed as soil multifunctionality, MF). The soil metrics included soil moisture, pH, organic carbon (OC), total nitrogen (TN), total P (TP), ammonium (NH_4_^+^) and nitrate (NO_3_^-^) contents, available phosphorous (AP) and potassium (AK) contents, microbial carbon (MBC) and nitrogen (MBN) contents, net mineralized organic carbon (Cm) and nitrogen (Nm). These variables provide information on a range of soil processes including nutrient availability, biogeochemical cycling and microbial productivity. Soil variables were measured using standard methods as described in Page et al. [48]. Soil moisture was measured by drying fresh soil samples at 105 °C to constant weight. Soil pH was measured in a soil: water (1:5) extract with a pH meter. Soil OC and TN were measured using the Walkley-Black and Kjeldahl method. Soil TP was measured by colorimetric analysis after digestion with sulfuric acid and perchloric acid. Soil NH_4_^+^ and NO_3_^-^ were measured using a continuous flow analyzer (AutoAnalyzer-AA3, Seal Analytical, Norderstedt, Germany) after extraction with 2 mol L^−1^ KCl. Soil AP was determined by the Olsen method. Soil AK was extracted with neutral ammonium acetate and measured by atomic absorption spectrometry (ZL-5100, PerkinElmer, MA). Soil MBC and MBN were measured using the fumigation extraction method [49] and a conversion factor of 0.45 and 0.54 was used to calculate MBC and MBN. The Cm and Nm was measured by incubating 10 g soil samples in jar at standard temperature (25 °C) for 28 days. Air samples were collected in days 0, 3, 7, 14, 21 and 28 after incubation for CO_2_ measurement with a CO_2_/H_2_O Analyzer (LI-6262, LI-COR Biosciences, Lincoln, NE). The total amount of CO_2_ released from soils was used to calculate the cumulative OC mineralized (Cm, mg CO_2_ kg^−1^). The net N mineralization (Nm, mg kg^−1^) was calculated as the difference of mineral N (NO_3_^-^ + NH_4_^+^) before and after the incubation. Soil pH is important in determining nutrients cycling and availability, however, because pH is a logarithmic scale, we did not include soil pH when calculating multifunctionality index.

Soil multifunctionality was assessed according to Fanin et al. [16]. In brief, data were tested for normal distribution by Shapiro-Wilk test prior to analyses. The non-normally distributed data were logarithm- or square root-transformed to make them close to normal distribution. For the variable that had negative values, we transformed the variable to be positive by subtracting the minimum value from the whole dataset. We then standardized the variables to the scale of 0 to 1, and take the average of these transformed values as multifunctionality values for each plot [16]. This method has been widely used [14, 16, 50, 51].

### Amplicon sequencing

Total DNA was extracted from soils by using FastDNA Spin Kits (MP Biomedical, Santa Ana, CA) as previously described [52]. The 515 F 5′-barcode- (GTGCCAGCMGCCGCGG)-3’ and 907 R 5′-CCGTCAATTCMTTTRAGTTT-3′ primers were used to amply 16 S rRNA gene at the hypervariable V4-V5 regions for bacterial and archaeal [53, 54]. Briefly, PCR was conducted in triplicate under the following conditions: 95 °C for 2 min, followed by 25 cycles at 95 °C for 30 s, 55 °C for 30 s, and 72 °C for 30 s and a final extension at 72 °C for 5 min. Sequencing libraries were prepared by using Illumina Nextera kit. Paired-end sequencing (2 × 250) was done by using an Illumina HiSeq 2500 platform (Shanghai BIOZERON Co., Ltd) at the Realbio Genomics Institute, Shanghai, China. Raw sequence data has been deposited in the sequence read archive at the N′CBI (https://www.ncbi.nlm.nih.gov/) under accession numbers PRJNA625503. The SHI7 program was used to trim and process the raw sequence reads by removing Nextera adapters, assembling sequences, and maintaining quality score via the threshold Q35 [55]. The trimmed sequences reads were further subjected to denoising, dereplication, and compaction by using NINJA-OPS program [56]. The high-quality sequence reads were aligned to Greengenes database 13_8 to obtain OTUs with 97% similarity by using Bowtie2 [57, 58]. More than 99.9% of our sequence reads were mapped against the database. A total of 3,222,996 reads were obtained from 48 samples, ranging from 21,111 to 119,141 reads per sample.

### Statistical analysis

Alpha diversity indices including Shannon index, observed species, and abundance-based coverage estimate (ACE) were calculated by using Mothur software8. Beta diversity among various erosion levels was evaluated by using Mothur with analysis of similarity (ANOSIM) function through Bray-Curtis dissimilarity matrices [59], and the P values were adjusted by Bonferroni correction as default in *anosim* function. Ordination of Bray-Curtis distance was performed by using Constrained Principle Coordinate Analysis (CPCoA) through the capscale function implemented in the vegan R library [60, 61]. ANOVA test via 10,000 permutations was used to calculate statistical significance.

Co-occurrence networks were constructed by using the WGCNA package based on the Spearman’s correlation matrices of Fuxian and Nenjiang [62]. Only OTUs with relative abundance >0.01% were used in the analyses. In networks, OTUs represent nodes, while correlations between OTUs are displayed as edges. Random matrix theory (RMT) was achieved to identify the appropriate similarity of 0.84 and 0.86 as the thresholds for Fuxian and Nenjiang, respectively [63]. Benjamini and Hochberg false discovery rate (FDR) were used to adjust the *P* values (*P* < 0.05) in the correlation [64]. The network properties were obtained by using the igraph package [65]. The network topological characteristics of each sample were implemented in subgraph function via the igraph package as described by Ma et al. [66]. The parameters describing network topological characteristics used in this study included node number (the number of OTUs), edge number (the number of connections among all of the nodes), betweenness (the number of times a node acts as a bridge along the shortest path between two other nodes), and assortativity (the extent of nodes in a network associate with other nodes in the network). A combination of degree, closeness centrality, betweenness centrality, and transitivity was used to statistically identify microbial keystone OTUs [67]. In this study, OTUs with degree and weighted degree >6, closeness centrality >0.14, and betweenness centrality <0.05, and transitivity (clustering coefficient) >0.09 were selected as potential keystone taxa. Visualization of co-occurrence network was performed by using Gephi [68]. Potential function among microbiota in different erosions levels was predicted by using Functional Annotation of Prokaryotic Taxa (FAPROTAX) via the default settings on the basis of taxonomic information of microorganisms [69].

We conducted mixed effect analysis of variance (ANOVA) to test the effects of site and erosion intensity on soil properties, multifunctionality, alpha-diversity metrics, parameters of co-occurrence networks. Prior to the analysis, data normality and homoscedasticity of variances was tested, and log- or log(x + 1) transformation was performed when necessary. We conducted generalized linear mixed effect model (GLMM) to examine the effects of site and erosion intensity on relative abundance of bacteria at phylum and family levels, and relative abundance of potential keystone taxa. Both mixed effect ANOVA and GLMM allow us to examine whether the effect of erosion intensity vary with sites. The effect of transect/slope was included as random effect. Specifically, we conducted site independent non-parametric test (Kruskal-Wallis test) to examine the effect of erosion on relative abundance of bacteria at phylum and family levels, and keystone taxa at each site. The ANOVA was conducted with JMP 10.0 (SAS Institute, Cary). The GLMM and Kruskal-Wallis test were conducted in R environment (v3.6.3; http://www.r-project.org/). Post hoc comparison was conducted by either the Turkey’s (for ANOVA) or Duncan’s test (Kruskal-Wallis test). Because some phyla and families were not identified simultaneously at both sites, we firstly conducted site independent analysis to test the effects of erosion intensity on relative abundance of all the identified phyla and of families with relative abundances of >1% for each site (Tables S1–S2). Specifically, at the phylum level, we examined the dominate taxa (Proteobacteria, Actinobacteria, Acidobacteria, Bacteroidetes, and Gemmatimonadetes) that accounted for more than 85% of the relative abundance of all identified phyla across both sites. At the family level, prior site independent analysis showed that erosion intensity significantly affected Chitinophagaceae, Gaiellaceae Solirubrobacteraceae, Nocardioidaceae at Fuxian, and Comamonadaceae, Haliangiaceae, Acidobacteriaceae, Nocardioidaceae and Frankiaceae at Nenjiang (Table S2), thereby these 8 families were examined to assess the effect of erosion intensity and whether such effect vary with sites.

We used linear regression analysis to establish relationship of soil MF to alpha-diversity metrics, parameters of co-occurrence networks and relative abundance of keystone taxa. Pearson correlations were used to assess the relationships of alpha-diversity metrics to soil properties. The redundancy analysis (RDA) was used to evaluate relationships between relative abundance of bacteria at phylum and family levels to soil moisture and nutrients. The linear regression and Pearson correlation analyses were conducted using JMP 10.0 (SAS Institute, Cary). The RDA was done in R environment (v3.6.3; http://www.r-project.org/).

## Results

### Soil quality and multifunctionality

Soil erosion caused significant changes in soil edaphic properties and reduced soil multifunctionality at both sites; however, the effects of erosion were greater at Nenjiang than at Fuxian (Table 1). The OC and TN contents, quantities of most available nutrients, MBC and MBN, and soil moisture were lower in eroded plots than in non-eroded plots at both sites. The net mineralized OC and N were also lower in eroded than in no-eroded plots at the Nenjiang site, but not at the Fuxian site. Soil erosion was negatively related to multiple soil parameters (e.g., soil multifunctionality) and soil multifunctionality was lower in eroded plots (Table 1).

**Table 1:** Effects of soil erosion on soil properties in Fuxian and Nenjiang sites.

Site	Erosion	pH	Moisture	OC	TN	TP	NH_4_^+^	NO_3_^−^	AP	AK	MBC	MBN	Cm	Nm	MF
			%	g/kg			mg/kg								
Fuxian	E0	8.19(0.02) a	21.9(0.35) a	7.5(0.2) a	0.72(0.02) a	0.25(0.02) b	4.78(0.32) a	1.78(0.22) a	1.88(0.07) a	110.7(3.7) a	91.1(5.8) a	3.7(0.2) a	3.9(0.18) a	2.9(1.5) a	0.85(0.02) a
	EL	8.22(0.03) a	20.8(0.25) ab	5.5(0.5) b	0.57(0.05) ab	0.26(0.00) ab	4.95(0.30) a	1.19(0.13) ab	1.98(0.53) a	91.8(1.6) c	41.9(21.0) ab	2.0(0.5) b	4.1(0.18) a	2.5(0.8) a	0.75(0.03) b
	EM	8.23(0.03) a	20.6(0.26) b	4.9(0.5) b	0.47(0.05) b	0.27(0.00) ab	4.27(0.37) a	0.77(0.12) b	1.65(0.09) a	99.1(1.8) bc	26.4(11.9) b	1.2(0.3) b	4.0(0.05) a	2.6(0.3) a	0.72(0.02) b
	EH	8.24(0.03) a	20.8(0.33) ab	4.7(0.3) b	0.45(0.04) b	0.28(0.01) a	4.58(0.62) a	1.17(0.11) b	1.69(0.15) a	101.5(2.0) ab	17.6(8.0) b	0.9(0.2) b	4.1(0.17) a	1.7(1.1) a	0.72(0.02) b
Nenjiang	E0	5.74(0.09) a	28.4(1.02) a	26.0(1.2) a	1.97(0.09) a	0.35(0.02) a	7.15(1.01) a	2.38(0.22) ab	27.10(1.48) a	175.8(7.7) a	188.1(17.6) a	16.3(3.2) a	3.8(0.27) a	21.3(1.2) a	0.78(0.02) a
	EL	5.70(0.06) a	21.7(1.68) b	20.3(1.9) a	1.50(0.13) b	0.24(0.02) ab	7.65(0.54) a	2.67(0.40) a	32.50(7.02) a	151.9(8.4) ab	138.4(8.3) a	14.1(3.0) a	3.3(0.22) a	12.9(1.7) b	0.70(0.02) b
	EM	5.73(0.08) a	14.6(1.82) c	12.0(1.8) b	0.90(0.15) c	0.17(0.01) bc	7.65(0.82) a	1.35(0.07) b	28.81(3.46) a	151.5(5.1) ab	45.6(15.2) b	12.1(4.5) a	3.5(0.24) a	8.1(1.0 bc	0.58(0.02) c
	EH	5.64(0.11) a	8.5(0.79) d	8.4(0.6) b	0.65(0.05) c	0.12(0.01) c	5.88(0.55) a	1.17(0.43) b	26.85(8.16) a	122.6(8.8) b	31.7(8.5) b	12.7(2.7) a	2.9(0.16) a	4.7(1.2) c	0.51(0.03) c
F	E	41.6	0.1	39.7	38.5	8.7	1.3	8.7	0.2	10.3	32.7	0.8	1.0	21.9	35.2
	S	14.9	2978.8	229.5	147.8	10.1	36.5	14.1	87.8	150.0	37.3	49.3	22.6	129.3	59.9
	S × E	33.8	0.4	21.9	17.1	16.3	1.2	3.0	0.2	5.9	6.2	0.1	2.6	17.1	6.1
P	E	<0.0001	0.9545	<0.0001	<0.0001	0.0001	0.3044	0.0002	0.8814	<0.0001	<0.0001	0.4859	0.4247	<0.0001	<0.0001
	S	0.0004	<0.0001	<0.0001	<0.0001	0.0029	<0.0001	0.0006	<0.0001	<0.0001	<0.0001	<0.0001	<0.0001	<0.0001	<0.0001
	S × E	<0.0001	0.7234	<0.0001	<0.0001	<0.0001	0.3351	0.0415	0.9023	0.002	0.0015	0.987	0.068	<0.0001	<0.0001
R2		0.86	0.99	0.91	0.89	0.69	0.56	0.54	0.65	0.82	0.79	0.58	0.41	0.86	0.82
RMSE		0.02	0.16	2.53	0.20	0.05	1.40	0.61	9.99	14.05	32.14	5.84	0.00	2.84	0.05

*E0* non-erosion, *EL* lightly erosion, *EM* moderately erosion, *EH* heavily erosion, *OC* organic carbon, *TN* total nitrogen, *TP* total phosphorous, *NH*_4_^+^ ammonium, *NO*_3_^−^ nitrate, *AP* available phosphorous, *AK* available potassium, *MBC* microbial carbon, *MBN* microbial nitrogen, *Cm* accumulative mineralized OC, *Nm* net mineralized N, *MF* multifunctionality of soils, *S* site, *E* Erosion, *RMSE* root mean square error for the model. Means followed by the same letter within the same column for each site are not significantly different at *α* = 0.05 according to Tukey’s test. The value in the parentheses is the standard error of the mean. The freedoms of site, erosion and their interactions were 1, 3 and 3, respectively.

Values with the same lower case were not significant at *P* < 0.05 among erosion levels for each site.

### Soil microbial diversity and its contribution to multifunctionality

**Fig. 1:**
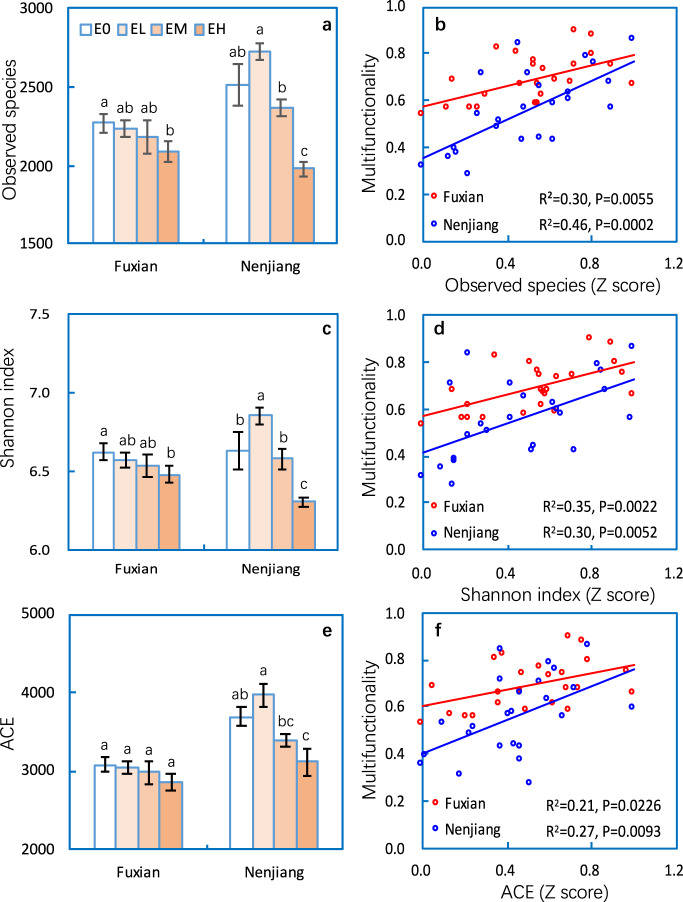
Alpha diversity of soil bacterial as affected by soil erosion and the relationships of soil multifunctionality to alpha diversity at the Fuxian and Nenjiang sites. Diversity of bacterial communities (Observed species, Shannon index and abundance-based coverage estimate (ACE)) in soils from non-eroded (E0), lightly eroded (EL), moderately eroded (EM) and heavily eroded (EH) plots (**a**, **c**, **e**), and the relationships of soil multifunctionality to diversity index (**b**, **d**, **f**) at the Fuxian and Nenjiang sites. Error bars are two standard errors of the mean. Means with the same lower case were not significant at *P* < 0.05 among erosion levels for each site.

Erosion resulted in marked declines in soil microbial diversity with greater effects at the Nenjiang than the Fuxian site (Fig. 1a, c, e, Table S3). The Shannon index, ACE, and observed species numbers were significantly lower in the eroded plots than in non-eroded plots at both sites. Nevertheless, soil microbial diversity was significantly and positively correlated with MF at each site (*P* < 0.05, Fig. 1b, d, f), and also correlated positively with most of the individual soil properties (e.g., OC and TN contents, available nutrients, and MBC and MBN) (*P* < 0.05, Fig. S3). Therefore, losses of microbial diversity in eroded soils were associated with downward shifts in soil ecosystem functions.

**Fig. 2:**
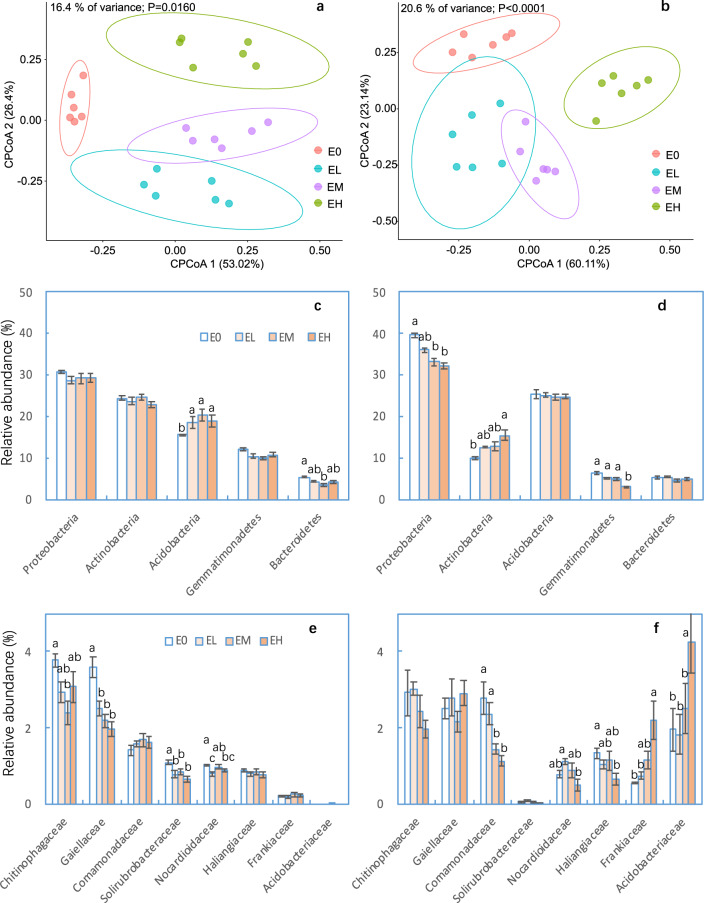
Soil bacterial composition as affected by soil erosion at the Fuxian and Nenjiang sites. Constrained Principle Coordinate Analysis (CPCoA) plot of Bray-Curtis distances among soil erosion treatments (**a**: Fuxian; **b**: Nenjiang), the relative abundance of the dominant bacterial phyla (**c**: Fuxian; **d**: Nenjiang) and of bacteria at family level that were significantly affected by soil erosion at any of the two sites (**e**: Fuxian; **f**: Nenjiang). The results of similarity comparison analysis among erosion treatments was significant at the Fuxian (*R* = 0.185, *P* = 0.025) and Nenjiang (*R* = 0.323, *P* < 0.001) sites (Table S2). The families less than 1% and that were not affected by erosion were not listed. E0: no n-eroded plots; EL: lightly eroded plots; EM: moderately eroded plots; EH: heavily eroded plots. Error bars are two standard errors of the mean. Means with the same lower case were not significant at *P* < 0.05 among erosion levels for each site.

Microbial community structure was also significantly affected by erosion and the concurrent changes in soil properties at both sites (Fig. 2). Soil community composition significantly differed between heavily eroded plots and non-eroded plots (*P* = 0.002) and between heavily eroded plots and lightly eroded plots (*P* = 0.004) at the Nenjiang site, and between non-eroded plots and moderately eroded plots (*P* < 0.001) and between non-eroded plots and heavily eroded plots (*P* = 0.004) at the Fuxian site, respectively (Table S4). Constrained principle coordinate analysis (CPCoA) showed that microbial communities strongly clustered according to soil erosion intensity, which explained ~16 and 21% of the total variation at the Nenjiang and Fuxian sites, respectively (Fig. 2a, b). These results indicated that soil erosion critically influenced the belowground microbiota.

### Soil microbial composition

Microbial communities in soils at both sites were primarily comprised of members of the phyla Proteobacteria, Actinobacteria, Acidobacteria, Bacteroidetes, and Gemmatimonadetes, accounting for ~85% of the relative abundance of all the identified phyla (Fig. S4). Erosion significantly decreased the relative abundance of Proteobacteria and Gemmatimonadetes at Nenjiang and Bacteroidetes at the Fuxian site (Fig. 2c, d, Tables S1 and S5). The abundance of Actinobacteria was significantly increased at Nenjiang, but slightly decreased at Fuxian. In contrast, the abundance of Acidobacteria was significantly increased at Fuxian, but only slightly decreased at the Nenjiang site (Fig. 2c, d, Tables S1 and S5).

At the family level, about 57–63% and 29–41% of the bacteria were not identified at the Fuxian and Nenjiang sites, respectively (Fig. S4). Consequently, we first focused on the response of bacteria with relative abundances of >1% to soil erosion, which varied by sites. At the Fuxian site, erosion resulted in a significant decrease in the abundance of Chitinophagaceae, Gaiellaceae, Solirubrobacteraceae, Nocardioidaceae, but had no effect on other taxonomic members. At Nenjiang, erosion resulted in a significant decrease in the abundance of Comamonadaceae, Haliangiaceae, and Nocardioidaceae, and a significant increase in the Acidobacteriaceae and Frankiaceae, but had no effect on other taxonomic members (Fig. 2e, f, Tables S2 and S6).

We classified specific bacteria clades into functional groups based on analyses done using the FAPROTAX (Functional Annotation of Prokaryotic Taxa) program [69], specifically focusing on taxa involved in soil N cycling, which is important for agricultural soils. We identified six bacterial families belonging to four N cycling functional groups, i.e., aerobic ammonia oxidation (Nitrososphaeraceae), aerobic nitrite oxidation (Nitrospiraceae), denitrification (Hyphomicrobiaceae) and nitrogen fixation (Beijerinckiaceae, Acetobacteraceae and Rhodospirillaceae). Results from site independent Kruskal-Wallis test demonstrated that soil erosion significantly increased the abundance of Acetobacteraceae in Fuxian plots (*P* = 0.0347) and Beijerinckiaceae in Nenjiang plots (*P* = 0.0114, Fig. S5, Table S7). In addition, the abundance of these two families were negatively related with MF (*P* = 0.0623 and 0.0002, respectively, Fig. S1). In contrast, the abundance of the other members of the N cycling bacteria identified here were not significantly affected by erosion (Table S7).

### Soil microbial network complexity

**Fig. 3:**
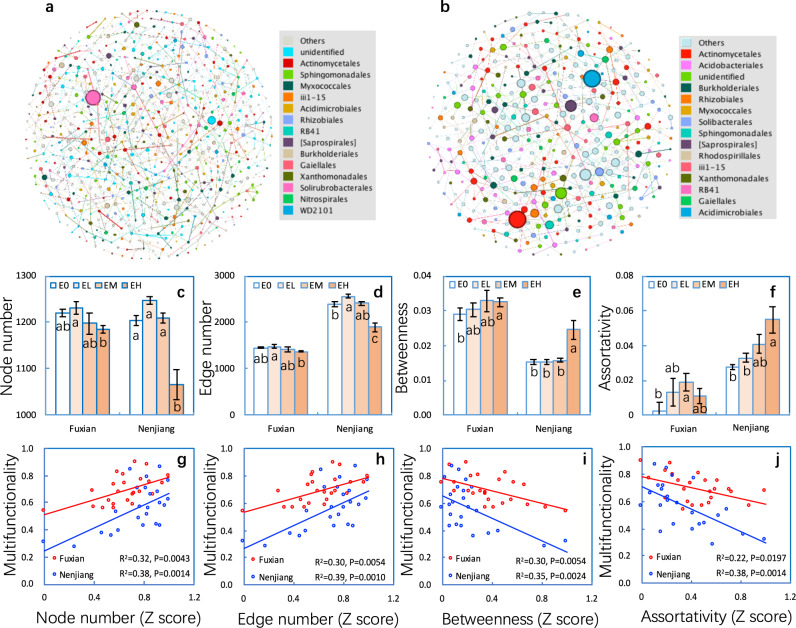
Co-occurrence patterns in soil bacterial as affected by soil erosion and the relationships of soil multifunctionality to co-occurrence assemblies at the Fuxian and Nenjiang sites. Co-occurrence network of soil bacterial at Fuxian (**a**) and Nenjiang (**b**). The sizes of the nodes (OTUs) are proportional to the number of connections. Only nodes (OTUs) that were significantly correlated each other (spearman’s > 0.84; after Benjamini and Hochberg FDR adjust, *P* < 0.05) were connected (edges). The numbers of node (**c**) and edge (**d**) and the degree of betweenness (**e**) and assortativity (**f**) of soil bacteria co-occurrence patterns from non-eroded (E0), lightly eroded (EL), moderately eroded (EM) and heavily eroded (EH) plots at Fuxian and Nenjiang. The relationships of soil multifunctionality to numbers of node (**g**) and edge (**h**) and the degree of betweenness (**i**) and assortativity (**j**) of soil bacteria co-occurrence patterns at the Fuxian and Nenjiang sites. Error bars are two standard errors of the mean. Means with the same lower case were not significant at *P* < 0.05 among erosion levels for each site.

The network of soil bacterial communities at each site demonstrated distinct co-occurrence patterns (Fig. 3a, b, Table S8). Here we used the network topological parameters of node and edge numbers, and betweenness and assortativity degree, to assess soil microbial network complexity, with higher node and edge numbers and smaller betweenness and assortativity representing greater network complexity. At both sites, the betweenness and assortativity were higher in the eroded plots than in non-eroded plots, whereas the node and edge numbers were lower. Moreover, such effects increased with erosion intensity (Fig. 3c–f). These results strongly suggest that soil erosion affected microbial associations, and thus reduced the complexity of soil microbial community networks. In addition, the changes in network complexity were strongly correlated (*P* < 0.05) with soil multifunctionality (Fig. 3g–j).

**Fig. 4:**
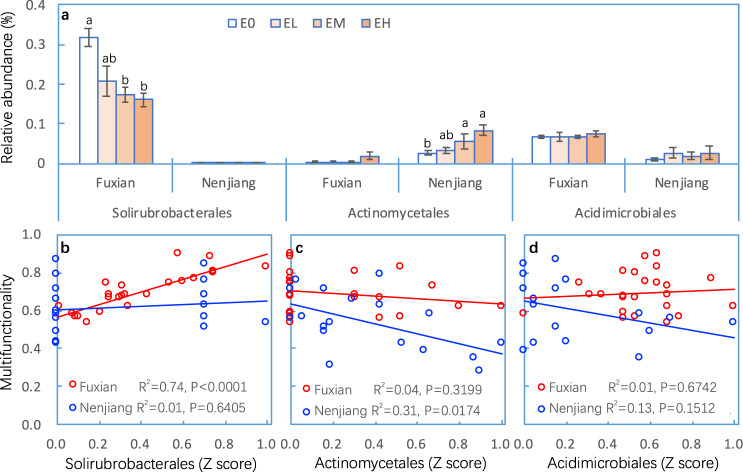
Relative abundance of keystone taxa as affected by soil erosion at the Fuxian and Nenjiang sites. Relative abundance of keystone taxa Solirubrobacterales, Actinomycetales and Acidimicrobiales identified from network analysis in soils from non-eroded (E0), lightly eroded (EL), moderately eroded (EM) and heavily eroded (EH) plots at the Fuxian and Nenjiang sites (**a**), and the relationships of soil multifunctionality to the abundance of Solirubrobacterales (**b**), Actinomycetales (**c**) and Acidimicrobiales (**d**). Error bars are two standard errors of the mean. Means with the same lower case were not significant at *P* < 0.05 among erosion levels for each site.

Network analysis also identified the Actinomycetales and Acidimicrobiales (both members of the class Actinobacteria) as keystone OTUs at the Nenjiang site, and the Solirubrobacterales (class Thermoleophilia) at the Fuxian site. The relative abundance of the Solirubrobacterales keystone OTU was positively correlated with MF, but decreased with the intensity of soil erosion at the Fuxian, but not at the Nenjiang site (*P* < 0.0001, Fig. 4a, b, Tables S9–S10). In contrast, while the relative abundance of the Actinomycetales keystone OTU increased with intensity of soil erosion and was negatively correlated with MF at the Nenjiang site, but not at Fuxian (Fig. 4a, c, Tables S9–S10), the Acidimicrobiales keystone OTU was neither affected by erosion intensity nor correlated with MF at either site.

## Discussion

### Erosion reduces microbial diversity and soil multifunctionality

Our examination of two sites with contrasting soil texture, climate, and erosion history revealed a consistent erosion-induced decrease in soil biodiversity and functionality. It has been well established that soil erosion results in the deterioration of soil structure, the loss of nutrients, decreases in the availability of nutrients, reduced water availability and a decrease in soil functionality [8–13, 70]. However, the impact of erosion on soil microbial communities has received less attention [43], and it was still unknown if erosion induced changes in soil microbial network complexity is linked to reduced soil multifunctionality. The decrease in soil organic matter, nutrients and moisture by erosion (Table 1) could have directly caused the loss of bacterial diversity and functionality because decreased resource availability constrains the metabolism and composition of bacteria [71–73] and thus impairs their supports on soil functionality [15, 31]. In addition, increased bulk density and decreased soil organic matter content in eroded soils can cause an increase in soil thermal conductivity and a decrease in soil heat capacity [74], resulting in greater daily and seasonal variation in soil temperature. Although not measured in this study, previous studies have shown greater variation in soil temperature at eroded sites than at non-eroded sites [75, 76]. Thus, the loss of diversity and functionality of soil communities can indirectly be attributed to an increase in soil thermal variability, because most soil microbes are sensitive to local changes in temperature [28, 77–79].

### Shifts in composition of soil bacteria

At the phylum level, we found decreases in the relative abundances of Proteobacteria, Bacteroidetes and Gemmatimonadetes by erosion. The Proteobacteria was the most abundant phylum in most of the soil samples collected in this study. Many Proteobacteria are considered copiotrophic, having relatively fast growth rate and capability to use various substrates [80–82]. The Bacteroidetes were previously reported to be a sensitive biological indicator for agricultural soil usage, with a significant decrease in arable soils than in wastelands [83]. The relative abundance Gemmatimonadetes was reported to be positively correlated with soil nutrients [84] (Fig. S6). Therefore, decrease in the relative abundances of these three phyla found in this study may be related to erosion-induced loss of available substrates and nutrients.

We also found that the abundance of Actinobacteria increased at Nenjiang and Acidobacteria increased at Fuxian. Members of Actinobacteria are sensitive to soil water conditions and negatively correlated with soil moisture [85–89]. In our study, erosion significantly decreased soil moisture at Nenjiang, but had minimum impact at the Fuxian site, which might have influenced the abundance of Actinobacteria in the Nenjiang plots, but not those at Fuxian. This explanation is consistent with the previous reports showing the decrease in the abundance of soil Actinobacteria caused by the addition of water [90]. Acidobacteria are considered to be oligotrophs and are also restricted by soil moisture, having negative correlations with most soil nutrients but a positive correlation with soil moisture [89, 91, 92] (Fig. S6). Consequently, our results suggest that the negative effects of decreasing soil moisture likely offset the positive effects of decreasing soil nutrients in influencing the abundance of Acidobacteria at the Nenjiang site, leading to a lack of response of this phylum to erosion. In contrast, at the Fuxian site, the lower nutrient content, but less of a decrease in soil moisture in eroded plots likely resulted in an elevated abundance of Acidobacteria.

Results of this current study showed that there were significantly lower relative abundances of some bacterial families in the eroded plots than in the non-eroded plots (e.g., Chitinophagaceae, Gaiellaceae, Solirubrobacteraceae and Nocardioidaceae at the Fuxian site, and Comamonadaceae, Haliangiaceae and Nocardioidaceae at the Nenjiang site). This response pattern is likely due to the dependency of these taxa on higher levels of soil moisture and nutrients as shown in Fig. S7 and in previous observations [73, 93, 94]. Interestingly, erosion resulted in an increase in members of the families Acidobacteriaceae and Frankiaceae at the Nenjiang site, most likely due to the negative impacts of soil moisture and nutrients on both families (Fig. S7). Moreover, we found a significant erosion-induced increase in the presence of the N fixing bacteria, Beijerinckiaceae and Acetobacteraceae, at both sites. This increase might be due to the reduction in N availability after erosion, which could make the environment advantageous for N fixing bacteria [95–97]. Further research is needed to confirm this supposition, including the comparison of N_2_ fixation rates between eroded and non-eroded soils. Overall, these results suggest that the shift in composition of bacteria after erosion was closely related with the response of soil conditions.

### Erosion affects microbiome complexity and keystone taxa

Our results also demonstrated that erosion reduced the network complexity of soil microbiomes at both sites. Reduced network complexity may result from enhanced resource limitation (e.g., reduced availability of water, soil carbon and nutrients) that impaired microbial diversity and network complexity [23, 35, 36, 98]. These results were consistent with previous observation that soil microbial network complexity increased with resource availability, such as soil fertility [99] and elevated CO_2_ [24], but decreased with reduced soil fertility [100], water availability [23] and intensified soil management in both agricultural [36] and forest [101] ecosystems.

Keystone taxa represent the highly connected microbes that play important roles in the structure and functions of microbiota and act as indicators of environmental changes [36, 39, 40], and hence are expected to have significant relationships to soil MF. In this study, we identified Solirubrobacterales (at Fuxian) and Actinomycetales and Acidimicrobiales (at Nenjiang) as keystone taxa, which were consistent with previous studies as keystone taxa in grassland, agricultural or desert soils [24, 39, 102–104]. While spatiotemporal heterogeneity drives the abundance and distribution of keystone taxa [39], the variations of keystone taxa in this study may result from the difference of pedogenic, adaphic, and climatic factors between these two sites.

That said, we did observe contrasting responses of keystone taxa to erosion between the two sites (Fig. 4a). The decrease in the relative abundance of Solirubrobacterales in response to erosion at Fuxian was expected because Solirubrobacterales was significantly and positively correlated with soil nutrients in previous studies [105, 106] as well as here (Fig. S8). This response pattern was consistent with the observations that the abundance of the class Thermoleophilia decreased significantly with increasing intensity of erosion at this site (*P* < 0.05, data not shown). The increases in the relative abundance of Actinomycetales in response to erosion at the Nenjiang site were consistent with the results showing that Actinobacteria (at the class level) increased with increasing erosion intensity (*P* < 0.05, data not shown). Most Actinobacteria favor aerobic environments [107], are adapted to arid conditions and are highly resistant to desiccation and low resource conditions [27, 41, 108]. The high clay and organic matter content in soils from Nenjiang may result in relatively high soil moisture content and potentially soils that are frequently anaerobic. In contrast, erosion often leads to the loss of OC and clay from soils, which decreases the ability of soil to retain water (Table 1), making the soils more aerobic, which could cause the increased abundance of the Actinobacteria. This may explain our observation that the keystone taxon Actinomycetales at the Nenjiang site were negatively correlated with soil OC and moisture (*P* < 0.05, Fig. S8). Similar to these results, previous studies have also reported that Actinobacteria was negatively related to soil moisture [41, 109], and has contrasting response patterns in responding to land-use change or exotic trees in forest ecosystem when compared with soil properties (i.e., organic matter, total C and N, available N) [110, 111]. Given the significant response to erosion and their relationships to MF (Fig. 4), as well as the important role in structure and function of microbiomes [24, 39, 102, 103], the keystone taxa identified herein (Solirubrobacterales at Fuxian and Actinomycetales at Nenjiang) could be used as an indicator of the effects of soil erosion and erosion intensity on microbial communities and soil multifunctionality.

### Variation in the effects of erosion between sites

The results presented here also show that erosion dramatically reduced soil multifunctionality, the diversity and network complexity of soil microbiota at both sites, suggesting that soil microbiota respond in the same way to erosion even in markedly different sites. Nevertheless, the effects of erosion were greater at the Nenjiang than at the Fuxian sites, likely due to the differences in erosion history, initial OC and N levels, and climate. Both sampling sites suffered erosion since their original conversion from forest, but the history of erosion was longer at Nenjiang (>50 years) than Fuxian (27 years). In contrast, soil OC and N were also closely related to the diversity and association among soil microbes [112–114]. The erosion-induced changes in OC and TN were also closely correlated to the changes in diversity and association of microbes when examined either across or within sites (Fig. S9). The greater initial or background concentrations of OC and N (present as the OC and TN contents in E0 plot) at the Nenjiang site, which was about threefold greater than that at Fuxian (Table 1). This corresponded to greater decreases in OC and TN after erosion [115] (Table 1), resulting in greater loss of diversity and associations among bacterial communities in eroded soils at the Nenjiang site than those at Fuxian.

Climate also likely has a major impact on soil functionality and microbiota [116–119]. In this study, we found that mean annual precipitation was similar at both sites (500 and 577 mm at Nenjiang and Fuxian, respectively), which might have similar impacts on rainfall-induced soil erosion. The higher mean annual temperature at Fuxian (9.0 °C) than at Nenjiang (0.4 °C) might have also resulted in larger effects of erosion on soil OC and N and microbiota because the decomposition of soil organic matter [115] and the diversity of soil community increased with temperature [116, 118, 119]. However, the lower concentration of OC and TN in Fuxian soils may have led to limitations in C and nutrients for microbial growth, restricting the effects of erosion in this warm site. Hence, the effects of climate may have been smaller than the effects of erosion history and the initial concentrations of OC and TN on the response of soil functions and diversity to erosion.

### Implications

Our findings have important implications for understanding the impact of soil erosion on the structure and function of soil microbial communities and how soil degradation is associated with specific soil microbiota. Erosion induced decreases in diversity and complexity of soil microbial communities at the two divergent sites. Importantly, soil microorganisms have essential supports on soil multifunctionality in diverse ecosystems by enhancing decomposition and nutrient cycling as well as resources availability [15, 32, 33]. The strong association between changes in soil bacterial communities and networks and the erosion-induced changes in soil quality examined herein emphasized the importance of soil communities in supporting multifunctionality in eroding environments (and adverse consequences when they cannot). Moreover, since diverse and complex microbial communities are more resilient to environmental stresses [26–28, 120, 121] than simple ones, a decrease in microbial diversity and complexity by soil erosion may have long-term adverse effects on soil functions. Therefore, post-erosion losses in soil multifunctionality and microbial diversity and complexity together can contribute to soil degradation and impair ecosystem services that the soil provides. The improvement of both aspects should be considered when rehabilitating degraded soils. Future research is needed to examine how degraded soil microbial communities and functions recover in response to erosion-prevention practices and mitigation strategies.

Network complexity and network topology as well as keystone taxa are assessed using statistical tools and are based on correlations [66, 67, 98]. Such correlations do not necessarily show cause-effect relationships. Although network complexity and keystone taxa play important role in microbial communities [23, 36, 38] and were significantly correlated with MF (Figs. 3 and 4), the present study did not directly examine such a role. Therefore, how they regulate the relationship between bacterial communities and erosion should be explicitly examined.

## Supplementary information

Supplementary Tables and Figs
