# Supplementary material for: Erosion reduces soil microbial diversity, network complexity and multifunctionality
Source: ISME J. 2021 Mar 12;15(8):2474–89. doi: 10.1038/s41396-021-00913-1 (PMC8319411; doi:10.1038/s41396-021-00913-1)
Supplement: Supplementary file 1 — Supplementary Tables and Figs [file 41396_2021_913_MOESM1_ESM.pdf]

Erosion Reduces Soil Microbial Diversity, Network Complexity and Multifunctionality

Liping Qiu[#], Qian Zhang[#], Hansong Zhu[#], Peter B. Reich, Samiran Banerjee, Marcel G.A. van der Heijden, Michael J. Sadowsky, Satoshi Ishii, Xiaoxu Jia, Mingan Shao, Baoyuan Liu, Huan Jiao, Haiqiang Li, Xiaorong Wei[*]

Supplementary information

Table S1 Results of Kruskal-Wallis test of the site independent effects of erosion (non-erosion, lightly erosion, moderately erosion and heavily erosion) on the relative abundance of taxa at phylum level at Fuxian and Nenjiang.

Fuxian			Nenjiang		
	Chisq	P		Chisq	P
Proteobacteria	0.55	0.9070	Proteobacteria	10.17	0.0172
Actinobacteria	0.45	0.9304	Actinobacteria	13.13	0.0044
Acidobacteria	10.98	0.0118	Acidobacteria	0.26	0.9674
Gemmatimonadetes	5.61	0.1324	Gemmatimonadetes	10.79	0.0129
Bacteroidetes	8.29	0.0380	Bacteroidetes	1.34	0.7197
Chloroflexi	2.06	0.5595	Chloroflexi	1.95	0.5821
Verrucomicrobia	1.57	0.6663	Verrucomicrobia	8.39	0.0386
Nitrospirae	3.48	0.3234	Nitrospirae	2.85	0.4159
Planctomycetes	3.12	0.3735	Planctomycetes	4.67	0.1979
Crenarchaeota	1.19	0.7562	Crenarchaeota	0.51	0.9174
TM7	4.65	0.1993	TM7	0.51	0.9170
Firmicutes	4.01	0.2602	Firmicutes	10.02	0.0184
WS3	3.40	0.3336	AD3	2.33	0.5074
			Armatimonadetes	5.15	0.1609

The freedom of erosion was 3.

Table S2 Results of Kruskal-Wallis test of the site independent effects of erosion (non-erosion, lightly erosion, moderately erosion and heavily erosion) on the relative abundance of taxa at family level.

Fuxian			Nenjiang		
	Chisq	P		Chisq	P
Chitinophagaceae	7.95	**0.0471**	Chitinophagaceae	5.97	0.1132
Gaiellaceae	13.63	**0.0035**	Gaiellaceae	2.26	0.5200
Comamonadaceae	2.17	0.5385	Comamonadaceae	15.39	**0.0015**
Solirubrobacteraceae	11.47	**0.0095**	Solirubrobacteraceae*	13.07	**0.0045**
Nocardioidaceae	11.38	**0.0099**	Nocardioidaceae	8.45	**0.0302**
Haliangiaceae	2.27	0.5182	Haliangiaceae	8.15	**0.0405**
Frankiaceae*	2.59	0.4601	Frankiaceae	11.46	**0.0095**
Acidobacteriaceae*	2.71	0.4392	Acidobacteriaceae	8.28	**0.0384**
Sphingomonadaceae	0.42	0.9361	Sphingomonadaceae	0.30	0.9600
Xanthomonadaceae	3.77	0.2876	Xanthomonadaceae	4.02	0.2598
Chthoniobacteraceae	2.57	0.4634	Chthoniobacteraceae	4.57	0.2062
Hyphomicrobiaceae	5.41	0.1440	Hyphomicrobiaceae	3.17	0.3657
Rhodospirillaceae	2.43	0.4887	Rhodospirillaceae	4.91	0.1787
Actinosynnemataceae	4.81	0.1859	Bradyrhizobiaceae	1.19	0.7557
Nitrososphaeraceae	1.19	0.7562	Caulobacteraceae	4.28	0.2326
Rubrobacteraceae	0.30	0.9600	Oxalobacteraceae	3.27	0.3523
Syntrophobacteraceae	2.68	0.4441	Koribacteraceae	0.72	0.8685
0319-6A21	1.80	0.6144	Solibacteraceae	4.33	0.2276

* The relative abundance of these taxa was smaller than 1%, and thus was not reported in main text and Fig. S*. The freedom of erosion was 3.

Table S3 Results of mixed effect variance analysis for the effects of sites (Fuxian and Nenjiang) and erosion (non-erosion, lightly erosion, moderately erosion and heavily erosion) on parameters of alpha diversity of soil microbiomes.

		Observed species	Shannon index	ACE
F	Erosion	13.0	8.4	7.0
	Site	14.5	0.7	40.7
	Site × Erosion	5.4	4.1	2.8
P	Erosion	<0.0001	0.0002	0.0007
	Site	0.0005	0.4013	<0.0001
	Site × Erosion	0.0033	0.0132	0.0519
R^2		0.625	0.478	0.626
RMSE		185.6	0.2	300.2

The freedoms of site, erosion and their interactions were 1, 3 and 3, respectively. RMSE: root mean square error for the model. ACE: abundance-based coverage estimate.

Table S4 The analysis of similarity comparison among erosion treatments on soil bacterial in Fuxian and Nenjiang sites.

Site	Fuxian		Nenjiang	
Comparison	R-value	P-value	R-value	P-value
E0-EL-EM-EH	0.185	0.025*	0.323	<0.001*
E0-EL	0.244	0.009	0.070	0.246
E0-EM	0.545	<0.001*	0.206	0.045
E0-EH	0.483	0.004*	0.667	0.002*
EL-EM	-0.052	0.556	0.113	0.152
EL-EH	-0.013	0.466	0.720	0.004*
EM-EH	-0.104	0.755	0.215	0.083

E0: non-erosion; EL: lightly erosion; EM: moderately erosion; EH: heavily erosion.

Table S5 Results of generalized linear mixed effect model for the effects of sites (Fuxian and Nenjiang) and erosion (non-erosion, lightly erosion, moderately erosion and heavily erosion) on the relative abundance of dominant taxa at phylum level.

	Erosion		Site		Site × Erosion	
	Chisq	P	Chisq	P	Chisq	P
Proteobacteria	9.13	0.0276	26.67	<0.0001	4.74	0.1917
Actinobacteria	3.11	0.3745	167.55	<0.0001	8.71	0.0334
Acidobacteria	2.66	0.4463	55.54	<0.0001	5.00	0.1716
Gemmatimonadetes	40.26	<0.0001	438.04	<0.0001	15.17	0.0017
Bacteroidetes	5.10	0.1647	2.25	0.1336	1.23	0.7465

The freedoms of site, erosion and their interactions were 1, 3 and 3, respectively.

Table S6 Results of generalized linear mixed effect model for the effects of sites (Fuxian and Nenjiang) and erosion (non-erosion, lightly erosion, moderately erosion and heavily erosion) on the relative abundance of taxa at family level which were significantly affected by erosion.

	Erosion		Site		Site × Erosion	
	Chisq	P	Chisq	P	Chisq	P
Chitinophagaceae	9.01	0.0292	3.37	0.0662	4.49	0.2128
Gaiellaceae	10.03	0.0182	0.02	0.8964	13.36	0.0039
Comamonadaceae	16.54	0.0009	6.03	0.0141	27.82	<0.0001
Solirubrobacteraceae	22.95	<0.0002	447.92	<0.0001	16.47	0.0009
Nocardioidaceae	9.29	0.0257	2.40	0.1216	17.06	<0.0001
Haliangiaceae	12.88	0.0049	6.71	0.0096	6.00	0.1117
Frankiaceae	25.52	<0.0001	52.27	<0.0001	23.96	<0.0001
Acidobacteriaceae	11.59	0.0089	85.44	<0.0001	11.59	0.0089

The freedoms of site, erosion and their interactions were 1, 3 and 3, respectively.

Table S7 Results of Kruskal-Wallis test of the site independent effects of erosion (non-erosion, lightly erosion, moderately erosion and heavily erosion) on the relative abundance of N cycling bacteria at family level.

	Fuxian		Nenjiang	
	Chisq	P	Chisq	P
Acetobacteraceae	8.63	0.0347	5.78	0.1228
Beijerinckiaceae	1.46	0.6904	11.06	0.0114
Hyphomicrobiaceae	5.41	0.1440	3.17	0.3657
Nitrososphaeraceae	1.19	0.7562	0.20	0.9773
Nitrospiraceae	3.75	0.2902	4.00	0.2615
Rhodospirillaceae	2.43	0.4887	4.91	0.1787

The freedom of erosion was 3.

Table S8 Results of mixed effect variance analysis for the effects of sites (Fuxian and Nenjiang) and erosion (non-erosion, lightly erosion, moderately erosion and heavily erosion) on parameters of network co-occurrence of soil microbiomes.

		Node number	Edge number	Betweenness degree	Assortativity degree
F	Erosion	20.1	25.8	5.2	4.3
	Site	6.3	724.8	116.8	50.7
	Site × Erosion	8.6	14.7	2.5	2.0
P	Erosion	<0.0001	<0.0001	0.0040	0.0100
	Site	0.0163	<0.0001	<0.0001	<0.0001
	Site × Erosion	0.0002	<0.0001	0.0766	0.1254
R^2		0.717	0.956	0.786	0.642
RMSE		37.589	113.319	0.004	0.013

The freedoms of site, erosion and their interactions were 1, 3 and 3, respectively. RMSE: root mean square error for the model.

Table S9 Results of generalized linear mixed effect model for the effects of sites (Fuxian and Nenjiang) and erosion (non-erosion, lightly erosion, moderately erosion and heavily erosion) on the relative abundance of keystone taxa identified in this study.

	Erosion		Site		Site × Erosion	
	Chisq	P	Chisq	P	Chisq	P
Actinomycetales	18.00	0.0004	40.20	<0.0001	6.00	0.1106
Acidimicrobiales	2.30	0.5109	58.70	<0.0001	0.70	0.8689
Solirubrobacterales	25.00	<0.0001	299.50	<0.0001	25.60	<0.0001

The freedoms of site, erosion and their interactions were 1, 3 and 3, respectively.

Table S10 Results of Kruskal-Wallis test of the site independent effects of erosion (non-erosion, lightly erosion, moderately erosion and heavily erosion) on the relative abundance of keystone taxa identified in this study.

	Fuxian		Nenjiang	
	Chisq	P	Chisq	P
Actinomycetales	4.11	0.2496	8.21	0.0419
Acidimicrobiales	0.81	0.8478	0.53	0.9124
Solirubrobacterales	10.25	0.0166	1.65	0.6473

The freedom of erosion was 3.

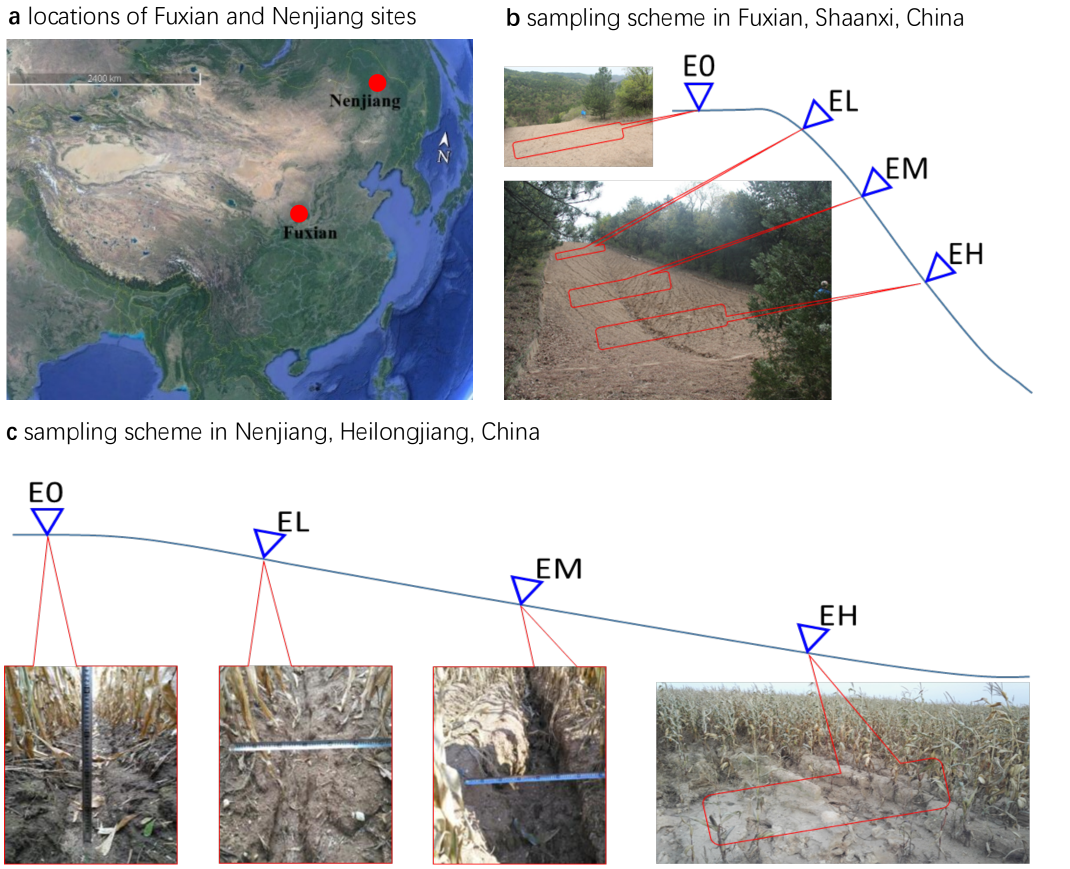

Fig. S1. The locations of Fuxian and Nenjiang sites (**a**) and sampling scheme at the Fuxian (**b**) and Nenjiang (**c**) sites. The averaged slope length and degree were 81 m and 27.8º at the Fuxian site, and were 1873 m and 4.1º at the Nenjiang site, respectively. Each site included two slopes. E0: non-eroded plot; EL: lightly eroded plot; EM: moderately eroded plot; EH: heavily eroded plot.

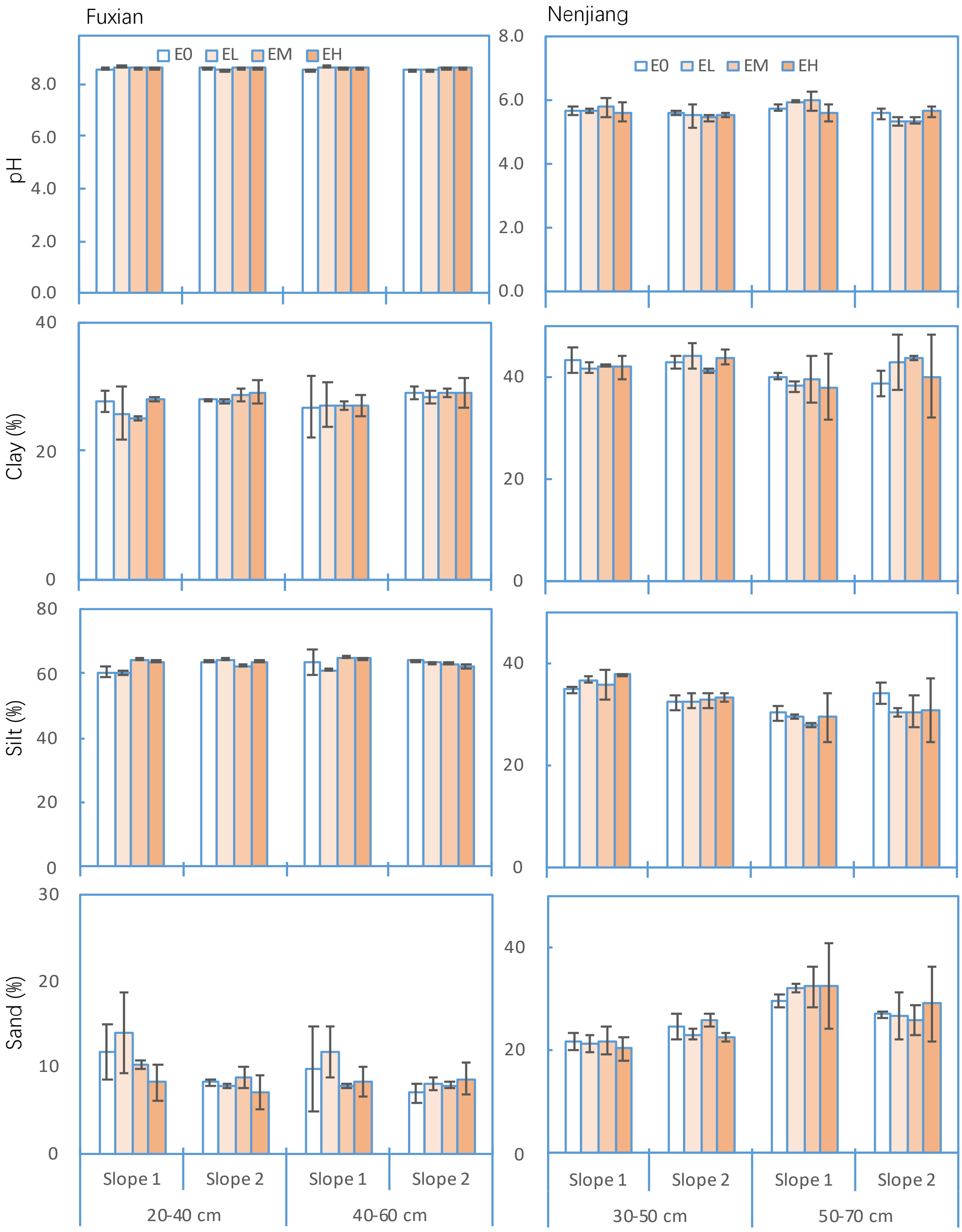

Fig. S2 The pH and contents of clay, silt and sand in deep soils (20-40 and 40-60 cm depth at Fuxian, 30-50 and 50-70 cm at Nenjiang) at each sampling position of each slope. E0: non-eroded plots; EL: lightly eroded plots; EM: moderately eroded plots; EH: heavily eroded plots. Error bars are two standard errors of the mean. P>0.05 for all the variables among each erosion treatments at each site and soil depth.

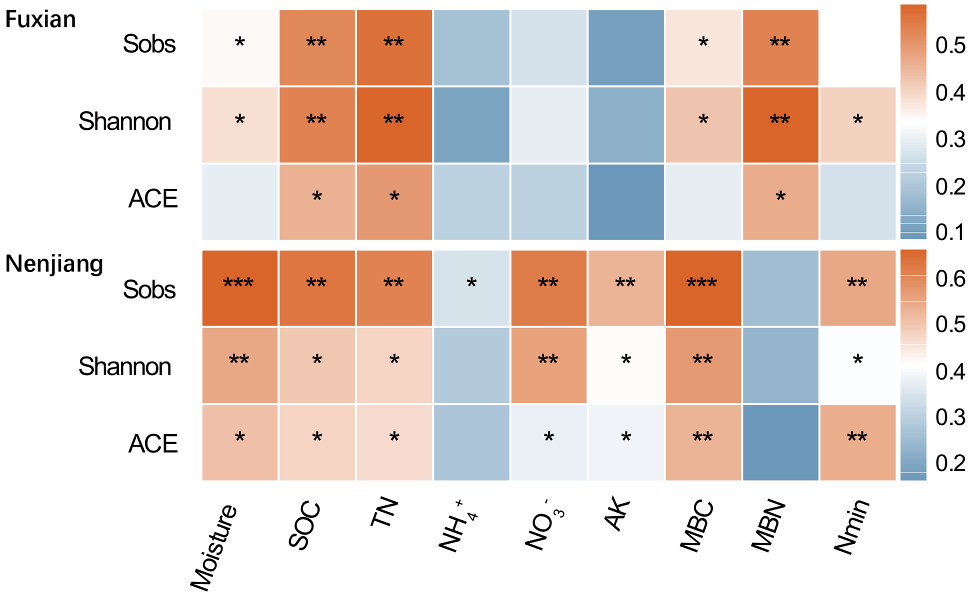

Fig. S3 The pearson correlationships of bacterial diversity (Observed species, Shannon index and abundance-based coverage estimate (ACE)) to soil variables at the Fuxian and Nenjiang sites, respectively. SOC: soil organic carbon; TN: total nitrogen; NH_4^+: ammonium; NO_3^-: nitrate; AK: available potassium; MBC: microbial biomass carbon; MBN: microbial biomass nitrogen; Nmin: net accumulative mineralized nitrogen. N=24 for each site. *: $0.01<P<0.05$; **: $0.001<P<0.01$; ***: $P<0.001$.

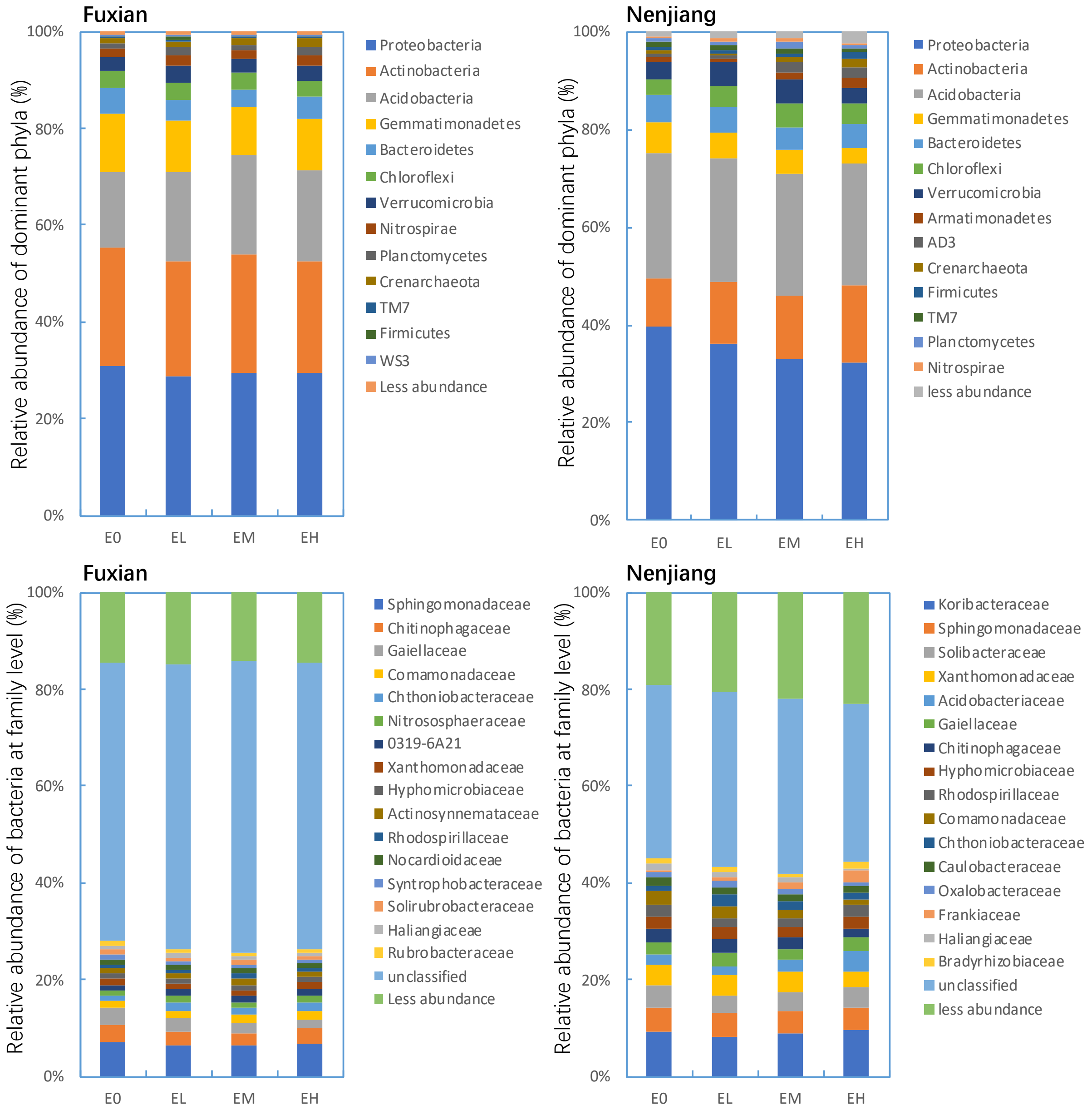

Fig. S4 Stacked bar chart showing the relative abundance of dominant phyla and that of bacteria at family level in soils from the Fuxian and Nenjiang sites. E0: non-eroded plots; EL: lightly eroded plots; EM: moderately eroded plots; EH: heavily eroded plots. The results of erosion on the relative abundance of taxa at phylum and family level either across or within site were presented in supplementary tables S1, S2, S5 and S6.

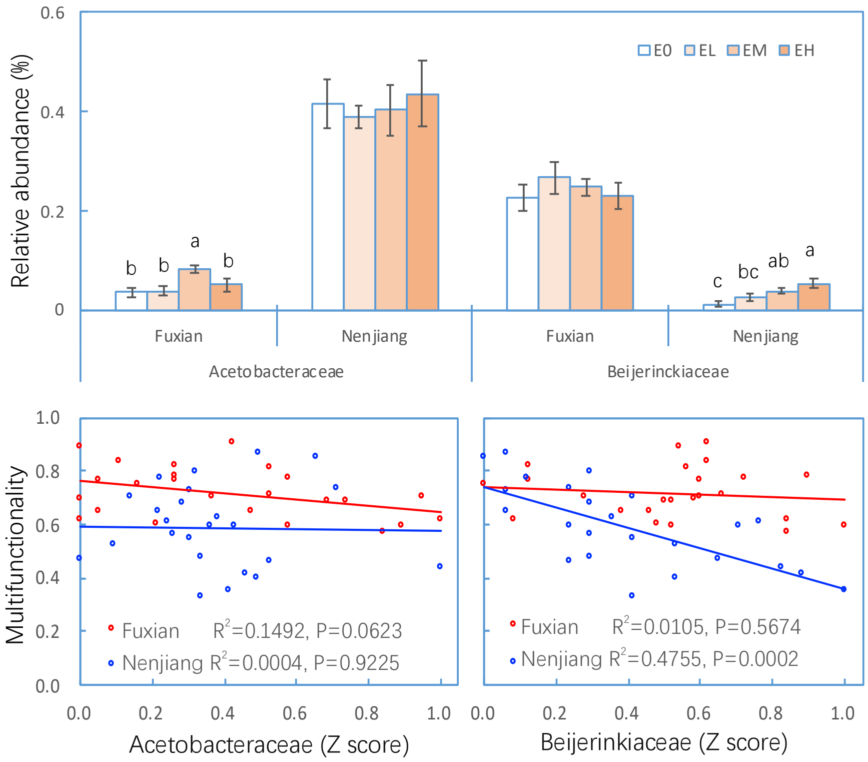

Fig. S5 Relative abundance of N cycling bacteria Acetobacteraceae and Beijerinkiaceae (at family level) in soils from non-eroded (E0), lightly eroded (EL), moderately eroded (EM) and heavily eroded (EH) plots at the Fuxian and Nenjiang sites and the relationships of soil multifunctionality to the abundance of these bacteria. Error bars are two standard errors of the mean. Means with the same lower case were not significant at $P<0.05$ among erosion levels for each site.

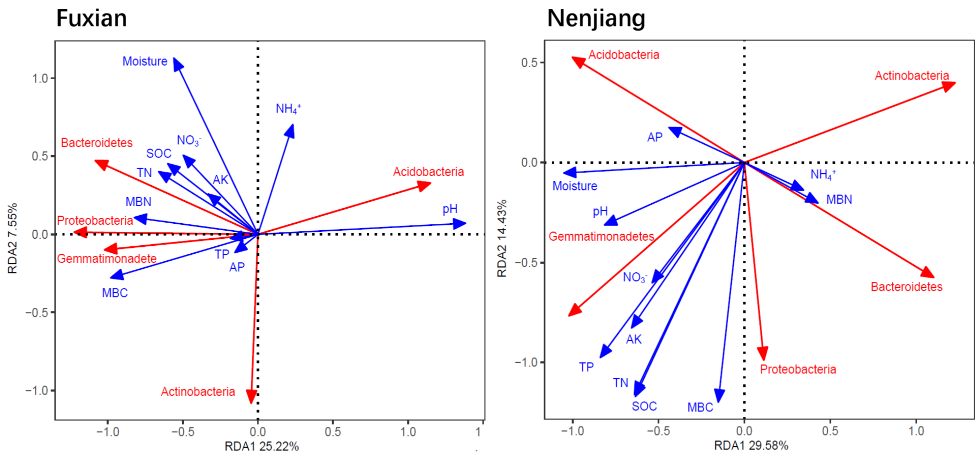

Fig. S6 The redundancy analysis (RDA) results identifying relationships of the relative abundance of dominant bacteria phylum to soil properties at Fuxian and Nenjiang. Moisture: soil moisture; pH: soil pH value; SOC: soil organic carbon; TN: total nitrogen; NH_4^+: ammonium; NO_3^-: nitrate; TP: total phosphorous, AP: available phosphorous; AK: available potassium; MBC: microbial biomass carbon; MBN: microbial biomass nitrogen.

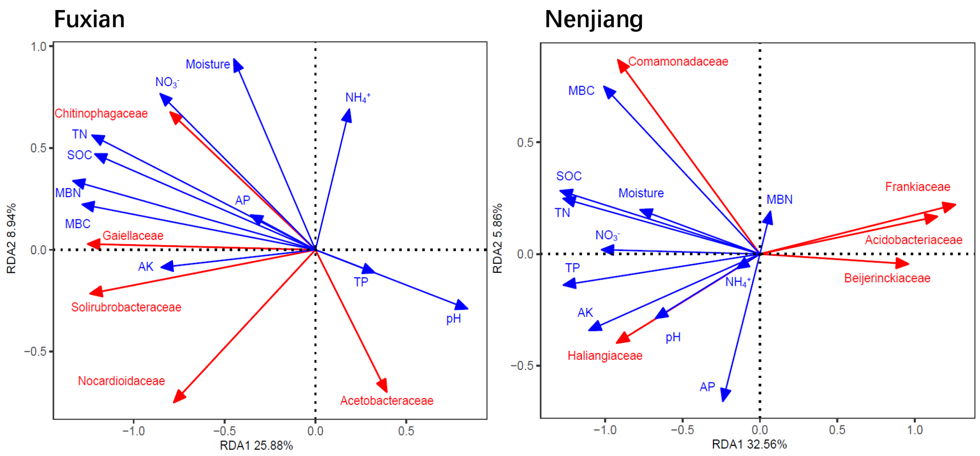

Fig. S7 The redundancy analysis (RDA) results identifying relationships of the relative abundance of bacteria at family level that were significantly affected by soil erosion to soil properties at Fuxian and Nenjiang. Moisture: soil moisture; pH: soil pH value; SOC: soil organic carbon; TN: total nitrogen; NH_4^+: ammonium; NO_3^-: nitrate; TP: total phosphorous, AP: available phosphorous; AK: available potassium; MBC: microbial biomass carbon; MBN: microbial biomass nitrogen.

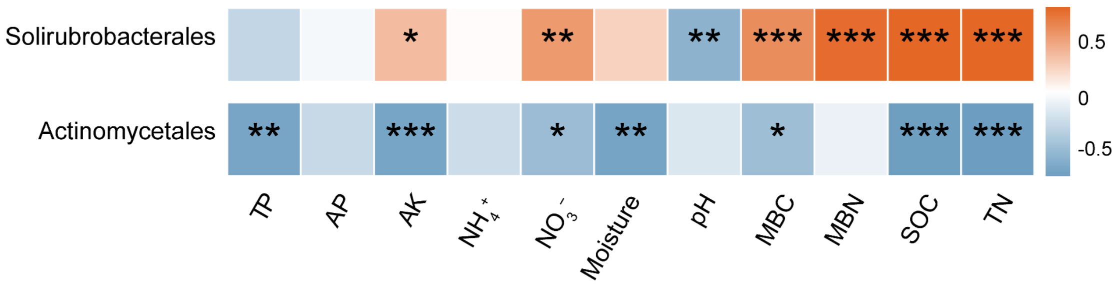

Fig. S8 The pearson correlationships of relative abundance of Solirubrobacterales at Fuxian and Actinomycetales at Nenjiang to soil variables, respectively. SOC: soil organic carbon; TN: total nitrogen; NH_4^+: ammonium; NO_3^-: nitrate; AK: available potassium; TP: total phosphorous; AP: available phosphorous; MBC: microbial biomass carbon; MBN: microbial biomass nitrogen. *: 0.01<P<0.05; **: 0.001<P<0.01; ***: P<0.001.

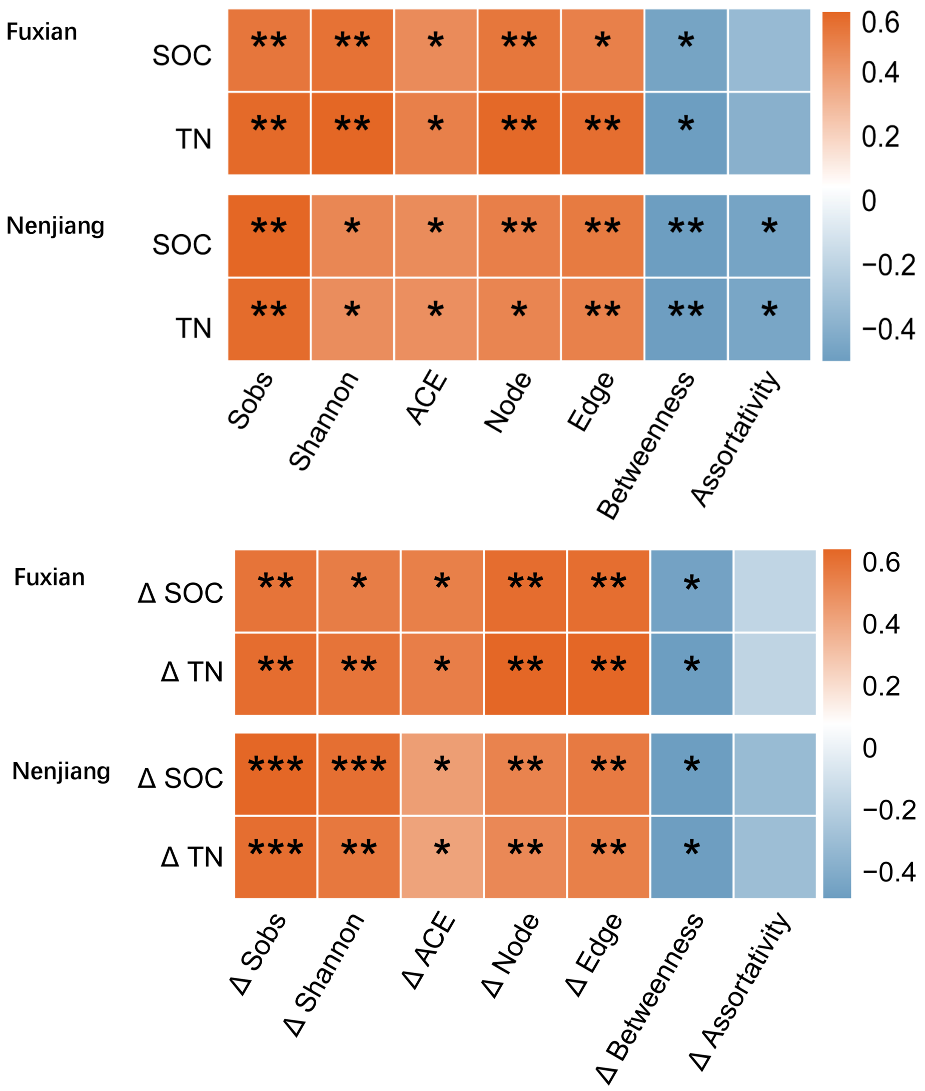

Fig. S9 The Pearson correlationships of bacterial diversity, co-occurrence network parameters and their changes after erosion to soil organic carbon, nitrogen and their changes after erosion at the Fuxian and Nenjiang sites, respectively. SOC: soil organic carbon; TN: total nitrogen; Sobs: observed species; Shannon: Shannon index; ACE: abundance-based coverage estimate; △: changes in these parameters. *: 0.01<P<0.05; **: 0.001<P<0.01; ***: P<0.001.
